# Predictors of chronic loneliness during adolescence: a population-based cohort study

**DOI:** 10.1186/s13034-022-00545-z

**Published:** 2022-12-21

**Authors:** Mariko Hosozawa, Noriko Cable, Syudo Yamasaki, Shuntaro Ando, Kaori Endo, Satoshi Usami, Miharu Nakanishi, Junko Niimura, Naomi Nakajima, Kaori Baba, Nao Oikawa, Daniel Stanyon, Kazuhiro Suzuki, Mitsuhiro Miyashita, Hiroyasu Iso, Mariko Hiraiwa-Hasegawa, Kiyoto Kasai, Atsushi Nishida

**Affiliations:** 1 Institute for Global Health Policy Research, Bureau of International Health Cooperation, National Center for Global Health and Medicine, 1-21-1 Toyama, Shinjuku-ku, Tokyo 162-8655 Japan; 2 Department of Pediatrics and Adolescent Medicine, Juntendo University, Tokyo, Japan; 3 Department of Epidemiology and Public Health, University College London, London, UK; 4 Research Center for Social Science and Medicine, Tokyo Metropolitan Institute of Medical Science, Tokyo, Japan; 5 Department of Neuropsychiatry, Graduate School of Medicine, The University of Tokyo, Tokyo, Japan; 6 Graduate School of Education, The University of Tokyo, Tokyo, Japan; 7 Department of Psychiatric Nursing, Tohoku University Graduate School of Medicine, Sendai, Japan; 8 Graduate School of Nursing Science, St. Luke’s International University, Tokyo, Japan; 9 Department of Community Mental Health, Shinshu University School of Medicine, Matsumoto, Japan; 10 Public Health, Department of Social Medicine, Osaka University Graduate School of Medicine, Osaka, Japan; 11 Department of Evolutionary Studies of Biosystems, The Graduate University for the Advanced Studies, Kanagawa, Japan; 12 The International Research Center for Neurointelligence, The University of Tokyo Institutes for Advanced Study, Tokyo, Japan

**Keywords:** Loneliness, Adolescence, Trajectory, Self-harm, Suicidal ideation, Bullying victimization, Parental psychological distress

## Abstract

**Background:**

Adolescent loneliness is a growing public health issue owing to its adverse health impact. Although adolescent loneliness is common, its trajectories can show distinct patterns over time. However, there is limited knowledge regarding their determinants, particularly for chronic loneliness. We aimed to determine the predictors of loneliness trajectories across early-to-mid adolescence and examine their association with later suicidality.

**Methods:**

Data were collected from 3165 participants from the population-based Tokyo Teen Cohort. Participants reported their loneliness at 10, 12, 14, and 16 years. Loneliness trajectories were identified using latent class growth analysis. We examined the predictive role of bullying victimization and parental psychological distress at age 10 via a multinomial logistic regression. Sociodemographic and child-related factors (i.e., chronic health conditions and cognitive delay) were included as covariates. The association between the trajectories, self-harm, and suicidal ideation by age 16 was investigated using Poisson regression.

**Results:**

Four trajectories were identified: “consistently low” (2448, 77.3%), “moderate–decreasing” (185, 5.8%), “moderate–increasing” (508, 16.1%), and “consistently high” (24, 0.8%). Taking “consistently low” as a reference, experiences of bullying victimization predicted all the remaining trajectories [adjusted relative risk ratio 1.64, 95% confidence interval (CI) 1.18–2.28 for “moderate–decreasing,” 1.88, 1.52–2.33 for “moderate–increasing,” and 4.57, 1.97–10.59 for “consistently high”]. Parental psychological distress predicted the “moderate–increasing” (1.84, 1.25–2.71) and “consistently high” (5.07, 1.78–14.42) trajectories. The “consistently high” trajectory showed the greatest risk for self-harm and suicidal ideation (adjusted relative risk ratio 6.01, 95% CI 4.40–8.22; 2.48, 1.82–3.37, respectively); however, the “moderate–increasing” and “moderate–decreasing” trajectories were also at increased risk (moderate–increasing: 2.71, 2.23–3.30 for self-harm, 1.93, 1.69–2.19 for suicidal ideation; moderate–decreasing: 2.49, 1.91–3.26 for self-harm, 1.59, 1.33–1.91 for suicidal ideation).

**Conclusions:**

Bullying victimization and parental psychological distress at age 10 were independent determinants of increased and chronic loneliness trajectories across early-to-mid adolescence. Compared with “consistently low,” all other loneliness trajectories were associated with an increased risk of adolescent suicidality. Interventions targeting adolescent loneliness should include approaches to mitigate bullying and parental psychological distress. These strategies may help prevent adolescent suicidality.

**Supplementary Information:**

The online version contains supplementary material available at 10.1186/s13034-022-00545-z.

## Background

Adolescence is a period characterized by dynamic biological, psychological, and social changes [1], making individuals vulnerable to experiencing loneliness [2]. Loneliness, defined as an unpleasant and distressing emotional state that arises from the discrepancy between desired and perceived social relationships [3], is particularly prevalent in adolescents [2]. For example, as many as 45% of 10–15-year-olds in the United Kingdom reported feeling lonely in 2018 [4]. Furthermore, the prevalence of loneliness among adolescents has increased dramatically in the last several decades across countries [5, 6]. Adolescent loneliness has been suggested as a significant risk factor for adolescent suicidality [7] and could lead to long-term mental health conditions in adulthood [8].

Although loneliness is common during adolescence, its trajectories can vary across individuals. Studies on the longitudinal patterns of adolescent loneliness have identified several distinct trajectories, including consistently low and consistently high [9–13]. In those studies, the consistently high loneliness trajectory was associated with various adverse health consequences [9, 10, 12, 13], indicating the importance of identifying predictors of chronic loneliness across adolescence. Existing research has mostly examined the predictive role of adolescents’ psychological or sociodemographic characteristics [9–13]. However, adolescent loneliness is reportedly associated with negative social relationships at school and home [14], and the relative contribution of heritability to loneliness is likely to decrease during early adolescence [15, 16]. This evidence suggests a need to investigate predictors other than an individual’s characteristics with regard to loneliness trajectories.

One potential predictor of adolescent loneliness, particularly chronic loneliness, is the experience of bullying victimization. Bullying often occurs among peers, and since adolescents increasingly rely on their peers for intimacy and support [17, 18], experiencing bullying victimization may lead to increased feelings of loneliness. Previous studies have shown an association between bullying victimization during adolescence and loneliness in adolescence [14] or young adulthood [19, 20]. However, whether experiences of bullying victimization in early adolescence predict chronic loneliness across adolescence remains unknown. Another potential predictor is parental psychological distress, which could increase adolescents’ loneliness through its adverse impact on parent–child relationships [21, 22]. However, to the best of our knowledge, no previous studies have examined the association between parental psychological distress and adolescent loneliness trajectories. Investigating the role of bullying victimization and parental psychological distress in adolescent loneliness trajectories, particularly chronic loneliness, which could lead to later suicidality, could have important implications for developing effective measures to tackle adolescent loneliness and associated health consequences.

Using data from a population-based cohort of adolescents from contemporary Tokyo, we aimed to (1) identify the trajectories of loneliness from early-to-mid adolescence (ages 10–16) and the role of experiences of bullying victimization and parental psychological distress in these trajectories and (2) examine the association between loneliness trajectories and suicidality (self-harm and suicidal ideation) by age 16. Based on previous evidence, we hypothesized that there would be several distinct adolescent loneliness trajectories, including consistently low and consistently high groups, associated with bullying victimization and parental psychological distress at different levels. In particular, we hypothesized that bullying victimization and parental psychological distress would predict the consistently high loneliness trajectory. We also hypothesized that the magnitude of the association between the trajectories of adolescent loneliness and suicidality would differ according to the different trajectories identified.

## Methods

### Participants

The Tokyo Teen Cohort (TTC) is an ongoing population-based cohort study following the physiological and psychological development of 3171 children born in three municipalities in the metropolitan area of Tokyo, Japan, between 2002 and 2004. A detailed description of the TTC has been provided elsewhere [23]. Surveys were conducted when the participants were aged 10, 12, 14, and 16 years. We restricted our sample to those who had valid responses for loneliness in at least one of the four study waves. Thus, after excluding six individuals, 3165 participants were included. All study procedures were approved by the Institutional Review Boards of the Tokyo Metropolitan Institute of Medical Science (approval number: 12-35), SOKENDAI (Graduate University for Advanced Studies, 2012002), and the University of Tokyo (10057). Written informed consent was obtained from all parents of the participating children, and informed assent was obtained from all children.

### Measurements

#### Loneliness

Loneliness was measured at ages 10, 12, 14, and 16 years using a single item from the Short Mood and Feelings Questionnaire. Participants were asked to respond to the statement “I felt lonely” with regard to the previous 2 weeks [24]; the response options were “not true,” “sometimes,” or “true.” This single-item measure correlates well with multi-item measures, such as the University of California Los Angeles Loneliness Scale [25, 26]. We treated loneliness as a three-level ordered categorical variable in our analysis.

#### Experiences of bullying victimization and parental psychological distress at age 10

We included experiences of bullying victimization and parental psychological distress measured in the age 10 survey as our predictors. The child’s experience of bullying victimization was identified through the following questions: “In the past 2 months, have you ever been bullied by other children in your school?” and “In the past 2 months, have you ever been bullied by other children outside of school?” The response options for both questions were “several times a week,” “about once a week,” “two or three times a month,” “one or two times in 2 months,” and “never.” Children who reported a frequency of “one or two times in 2 months” or higher to either question were classified as having experienced bullying victimization at age 10. Parental psychological distress was assessed with the Kessler Psychological Distress Scale (K6 +), a validated measure of psychological distress used to evaluate symptoms of depression and anxiety in the past 30 days [27]. The scale included six items (e.g., “During the past 30 days, how often did you feel nervous?”) rated from 0 (“None of the time”) to 4 (“All of the time”). Higher scores indicated more severe psychological distress (Cronbach’s alpha 0.84). Respondents’ parents who scored above 10 were classified as having experienced psychological distress according to the cut-off used in Japanese national statistics [28].

#### Self-harm and suicidal ideation by age 16 years

Self-harm was assessed by the question, “Have you ever intentionally hurt yourself in the past year?” asked at ages 12, 14, and 16 years. Children who answered “yes” in any of the waves were classified as having a history of self-harm by the age of 16. Suicidal ideation was assessed at age 16 by asking, “Have you ever wanted to die?” Children who answered “yes” were identified as having suicidal ideation by age 16.

#### Covariates

We included the following sociodemographic and child-related variables that have been reported to be associated with loneliness as covariates [13, 14, 29, 30]. The child’s sex was defined as “boy” or “girl” based on parental report. Parental origin was classified as both parents being Japanese or not. Low household income was indicated by an annual household income below 4,000,000 yen (approximately USD $30,000), just below the median national income in Japan. Parental education was identified based on either a higher or lower qualification than high school. Parenthood was grouped as being a single parent or not. A chronic health condition was identified through the question, “Does the child have any physical or mental health conditions or illnesses lasting or expected to last 12 months or more?” The child’s cognitive ability was assessed through an interview that used the short form of the Wechsler Intelligence Scale for Children, where those who scored below 85 were classified as having a cognitive delay [31]. All the covariates were measured at age 10, and apart from the child’s cognitive ability, all data for covariates were reported by the parent/s.

### Statistical analyses

First, we examined the descriptive characteristics of the participants and the proportion of each loneliness category at each wave. Subsequently, to identify variations in the trajectory of loneliness across early adolescence, we conducted a latent class growth analysis using data on loneliness measured at ages 10, 12, 14, and 16 as a three-level ordered categorical variable. We fitted models with two to five trajectories, informed by past studies on adolescent loneliness trajectories [9–13]. The best-fitting model was identified using the Akaike information criterion, sample adjusted Bayesian information criterion, entropy index, Vuong–Lo–Mendell–Rubin test, and clinical utility of the model [32]. A detailed description of the trajectory modeling is presented in Additional file 1: Table S1.

Once the best-fitting trajectory model was identified, we examined the predictors for each loneliness trajectory using multivariable multinominal logistic regressions, adjusting for all predictive variables. Finally, we examined the association between group memberships of the trajectories and suicidality by age 16 using generalized linear models with Poisson distribution and robust standard errors. A crude model (Model 1), a model adjusted for the covariates measured at age 10 (child’s sex, parental origin, low parental education, low household income, parenthood, child’s chronic health condition, and child’s cognitive delay; Model 2), and a fully adjusted model, further adjusted for experiences of bullying victimization and parental psychological distress (Model 3), were examined. As a sensitivity analysis, we repeated the latent class growth analysis and multivariable multinominal logistic regressions and restricted our sample to those with valid loneliness responses for more than two points (n = 2813). Data on loneliness were available for 3136 (99.1% of our sample) participants at age 10; 2520 (79.6%) participants at age 12; 2105 (66.5%) participants at age 14; and 2050 (64.8%) participants at age 16. Missing data were handled with full information maximum likelihood in the latent class growth analyses. Thereafter, we conducted multiple imputation by chained equations on our main predictors and covariates and included the identified group membership and auxiliary variables in the imputation model. The proportion of missing data ranged from 0.06% for cognitive delay to 38.4% for suicidal ideation.

Regression analyses were run across 40 imputed datasets and adjusted using Rubin’s rules [33]. We presented the imputed results (imputed on our main predictors and covariates) as they were broadly similar to those obtained using the observed cases (Table 1 and Additional file 2: Table S2).

**Table 1:** Descriptive characteristics of the observed sample (N = 3165)

	n	%
*Gender*		
Boy	1680	53.1
Girl	1485	46.9
*Parental origin*		
Japanese	3093	97.7
Non-Japanese	72	2.3
*Low parental education*^*a*^		
No	2633	83.3
Yes	529	16.7
*Low household income*^*b*^		
No	2717	89.4
Yes	324	10.7
*Single-parent household*		
No	3007	95.0
Yes	158	5.0
*Child chronic health condition*		
No	2829	89.6
Yes	329	10.4
*Child cognitive delay*^*c*^		
No	3013	95.3
Yes	150	4.7
*Bullying victimization*		
No	2313	73.4
Yes	840	26.6
*Parental psychological distress*^*d*^		
No	2990	95.0
Yes	157	5.0

N varies owing to missing values. All variables were measured at age 10 (baseline)

^a^Defined as the respondent parent having completed a higher or lower qualification than high school

^b^Defined as a household income below 4,000,000 yen (approximately $30,000)

^c^Defined as intelligence quotient below 85

^d^Defined as scoring above 10 on the Kessler Psychological Distress Scale

Approximately half (n = 1184) of the participants in the age 16 wave were interviewed during the coronavirus disease 2019 pandemic (i.e., after March 2020), which may have impacted their response to loneliness. The proportion of those who reported any loneliness at age 16 was slightly higher for adolescents interviewed during the pandemic (16% before pandemic vs. 21% during pandemic). However, there was no significant difference in the proportion of adolescents interviewed before and during the pandemic in each trajectory. Thus, the two groups were analyzed together. Latent class growth analyses were conducted using Mplus 8.7, and all other analyses were conducted using Stata SE version 17 (StataCorp, College Station, TX, USA). We followed the Strengthening the Reporting of Observational Studies in Epidemiology guidelines for reporting the results.

## Results

**Fig. 1:**
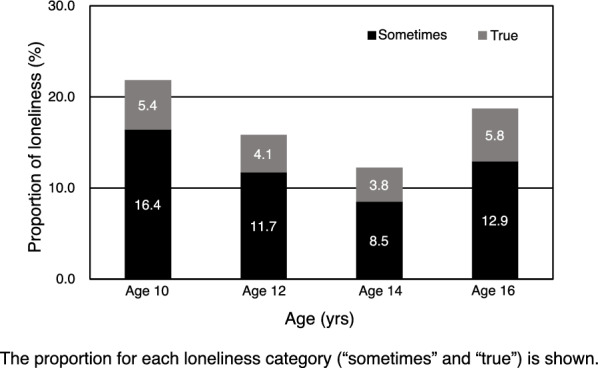
Proportion of loneliness at each age wave. The proportion for each loneliness category (“sometimes” and “true”) is shown

Of the 3165 adolescents in our sample, 1485 (46.9%) were girls. Of these, one-fourth (n = 840, 26.6%) reported experiencing bullying victimization at age 10. In addition, parental psychological distress was observed in 157 (5%) respondents. Across waves, the proportion of adolescents who reported loneliness was highest at age 10 (21.8%) and decreased thereafter (Fig. 1). However, a slight increase was observed between ages 14 and 16 years. Approximately 5% of the adolescents reported frequent loneliness, as indicated by the “true” response in the loneliness question across the waves.

**Fig. 2:**
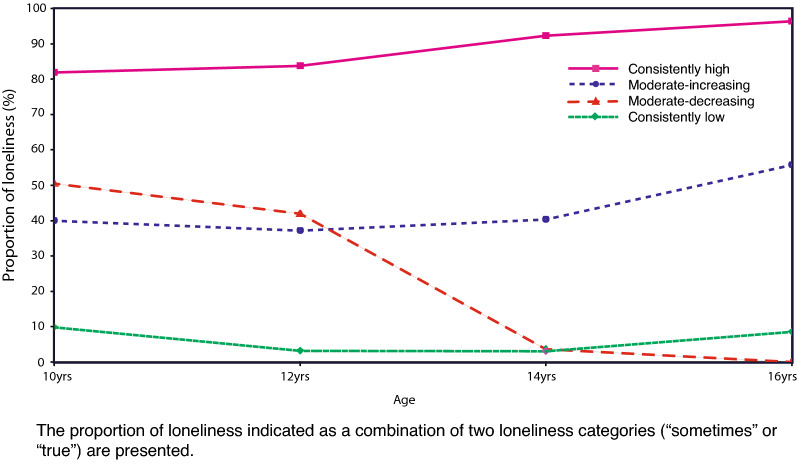
Proportion of loneliness by identified trajectories. The proportion of loneliness indicated as a combination of two loneliness categories (“sometimes” or “true”) is shown

The trajectory modeling resulted in four groups (Fig. 2, Additional file 3: Table S3). Most of the participants (n = 2448, 77.3%) belonged to the “consistently low” group, which indicated a low possibility of experiencing loneliness across early-to-mid adolescence. The “moderate–decreasing” group (185, 5.8%) had a moderate risk of experiencing loneliness at age 10, which decreased with age. For example, at age 10, 35.9% of the adolescents in this group felt lonely “sometimes,” and 14.6% frequently felt lonely. However, at age 16, all the participants in this group responded that they did not feel lonely (Additional file 4: Table S4). The “moderate–increasing” group (508, 16.1%) had a moderate risk of experiencing loneliness at age 10 (29.8% felt lonely “sometimes” and 10% frequently felt lonely), and this risk increased with age (38.2% felt lonely “sometimes” and 17.4% frequently felt lonely at age 16). The “consistently high” group was the smallest (24, 0.8%) and tended to experience frequent loneliness across early-to-mid adolescence, which resulted in 86.5% reporting frequent loneliness at age 16.

The predictors of trajectory group membership were identified through a multivariable multinominal logistic regression that took the “consistently low” group as a reference (Table 2; detailed characteristics of each loneliness trajectory group are shown in Additional file 2: Table S2). Experience of bullying victimization at age 10 predicted membership in all three trajectory groups: “moderate–decreasing” [adjusted relative risk ratio (aRRR) = 1.64, 95% confidence interval (CI) 1.18–2.28], “moderate–increasing” (aRRR 1.88, 1.52–2.33), and “consistently high” (aRRR 4.57, 1.97–10.59). Parental psychological distress when the child was aged 10 predicted membership in the “moderate–increasing” (aRRR 1.84, 1.25–2.71) and “consistently high” (aRRR 5.07, 1.78–14.42) groups.

**Table 2:** Adjusted relative risk ratios for predictors of adolescent loneliness trajectories^a^

	Moderate–decreasing (n = 185, 5.8%)			Moderate–increasing (n = 508, 16.1%)			Consistently high (n = 24, 0.8%)		
	RRR	95% CI		RRR	95% CI		RRR	95% CI	
Bullying victimization	1.64**	1.18	2.28	1.88***	1.52	2.33	4.57***	1.97	10.59
Parental psychological distress	0.67	0.29	1.56	1.84**	1.25	2.71	5.07***	1.78	14.42
Gender (girl)	1.42*	1.05	1.94	2.32***	1.89	2.84	2.01	0.87	4.63
Parent origin (non-Japanese)	0.82	0.25	2.68	1.98*	1.14	3.44	2.36	0.30	18.35
Low parental education	1.25	0.84	1.86	1.39**	1.08	1.79	1.24	0.46	3.37
Low household income	1.12	0.66	1.90	1.24	0.89	1.74	1.30	0.38	4.41
Single-parent household	0.95	0.46	1.97	0.86	0.53	1.39	0.91	0.17	4.88
Child chronic health condition	1.82**	1.19	2.77	1.31	0.96	1.78	1.22	0.39	3.79
Child cognitive delay	0.89	0.44	1.80	0.84	0.53	1.34	4.20**	1.44	12.24

*CI* confidence interval; *RRR* relative risk ratio

The “consistently low” group is taken as reference

*p < 0.05, **p < 0.01, ***p < 0.001

^a^ All predictive variables were mutually adjusted in the model

Table 3 shows the association between the identified trajectories and adolescent suicidality. The risk of self-harm by age 16 was most remarkable for the “consistently high” group [relative risk (RR) 6.78, 95% CI 5.10–9.02], followed by the “moderate–increasing” (RR 2.87, 2.37–3.48) and “moderate–decreasing” groups (RR 2.54, 1.94–3.32). A similar trend was observed for suicidal ideation; the RR was 2.61 (95% CI 1.90–3.58) for “consistently high,” 2.18 (1.93–2.47) for “moderate–increasing,” and 1.67 (1.39–2.00) for “moderate–decreasing.” Further adjusting for covariates did not change the association for both outcomes. Our sensitivity analysis, limited to adolescents with valid loneliness responses for more than two of four time points, yielded similar group trajectories and did not change the results (results available upon request).

**Table 3:** Relative risk of self-harm and suicidal ideation by age 16 by adolescent loneliness trajectories

Self-harm	Case/n^c^	Model 1: crude			Model 2: partially adjusted^a^			Model 3: fully adjusted^b^		
		RR	95% CI		RR	95% CI		RR	95% CI	
Consistently low	225/2154	1.00	–		1.00	–		1.00	–	
Moderate–decreasing	49/185	2.54***	1.94	3.32	2.51***	1.92	3.30	2.49***	1.91	3.26
Moderate–increasing	126/420	2.87***	2.37	3.48	2.79***	2.30	3.39	2.71***	2.23	3.30
Consistently high	17/24	6.78***	5.10	9.02	6.61***	4.90	8.91	6.01***	4.40	8.22
*Suicidal ideation*										
Consistently low	417/1483	1			1			1		
Moderate–decreasing	76/162	1.67***	1.39	2.00	1.61***	1.34	1.92	1.59***	1.33	1.91
Moderate–increasing	168/274	2.18***	1.93	2.47	1.96***	1.73	2.23	1.93***	1.69	2.19
Consistently high	11/15	2.61***	1.90	3.58	2.59***	1.92	3.51	2.48***	1.82	3.37

*RR* relative risk, *CI* confidence interval

^a^Adjusted for child’s gender, parental origin, low parental education, low household income, single parenthood, child chronic health condition, and child cognitive delay

^b^Adjusted for Model 2 + bullying victimization and parental psychological distress at age 10

^c^N varies owing to missing outcomes

## Discussion

In this population-based cohort, we identified four distinct loneliness trajectories among adolescents aged 10‒16 years. Across early-to-mid adolescence, most participants (77%) had a low risk of experiencing loneliness. Approximately one-fifth had a moderate risk of loneliness at age 10, which increased and decreased with age in two separate groups (16% and 6%, respectively). A substantial minority (0.8%) experienced chronic loneliness. Experience of bullying victimization at age 10 predicted membership in the “consistently high,” “moderate–increasing,” and “moderate–decreasing” loneliness trajectories. Parental psychological distress when the child was aged 10 predicted membership in the “moderate–increasing” and “consistently high” groups. The “consistently high” group was at greatest risk for suicidality by age 16 and showed a sixfold and threefold risk for self-harm and suicidal ideation, respectively. However, the “moderate–increasing” and “moderate–decreasing” groups were also at a two–three-fold risk for self-harm and suicidal ideation compared with the “consistently low” group.

The four trajectory patterns identified in our study echoed previous studies, where four to five distinct adolescent loneliness trajectories were observed [9, 11–13]. Similar to previous studies, the largest trajectory group in our study was adolescents who experienced low or no loneliness across adolescence, while the proportion of adolescents who experienced chronic loneliness was the smallest. In our study, the proportion of adolescents experiencing chronic loneliness (0.8%) was smaller than that reported in previous studies (ranging from 3 to 22%) [9, 11–13]. The smaller proportion of those with chronic loneliness in our study could be because of the use of a single-item measure of loneliness compared to multi-item measures used in previous studies [9, 11–13]; this is because a single item may show a lower prevalence of loneliness [34]. Alternatively, this result could be reflective of cultural differences in the prevalence of adolescent loneliness. A study from the UK using the same single-item measurement reported the prevalence of loneliness to be 34% among 14-year-olds, higher than the figure in our study (12% in the age 14 survey) [14]. Despite the differences in the proportion rate of each trajectory, the patterns identified based on a single-item measure of loneliness reflected existing evidence from Western Europe and North America, where multi-item indicators of loneliness were used [9, 11–13]. This may suggest that adolescent loneliness trajectories follow similar patterns regardless of culture.

Overall, our findings indicate that bullying victimization and parental psychological distress are independent determinants of adolescent loneliness, particularly for increased or chronic loneliness across early-to-mid adolescence. Experiences of bullying victimization at age 10 predicted all loneliness trajectories over the “consistently low” group and showed the strongest association with the “consistently high” group. Our findings are consistent with previous results that showed a positive association between childhood bullying victimization and loneliness in young adulthood [19, 20]. Furthermore, our results may expand upon the literature by indicating that experiencing bullying victimization at the beginning of adolescence is generally predictive of any adolescent loneliness trajectory, although its influence may be particularly strong for adolescents with chronic loneliness. In contrast, parental psychological distress measured at age 10 predicted the “moderate–increasing” and “consistently high” trajectories, but not the “moderate decreasing” trajectory. To our knowledge, our study is the first to examine the role of parental psychological distress in adolescent loneliness trajectories. Our results indicate that heightened parental psychological distress at the beginning of adolescence may amplify adolescents’ feelings of loneliness, possibly through unstable parent–adolescent relationships [21]. Given that bullying victimization and parental psychological distress could be modified or prevented through interventions [17, 35], our results underline the importance of considering these factors to reduce adolescent loneliness.

Consistent with a previous study [13], the “consistently high” trajectory was at the highest risk of later suicidality and showed a sixfold and threefold risk of self-harm and suicidal ideation by age 16, respectively. Since self-harm and suicidal ideation are significant risk factors for suicide attempts [36], our results highlight that adolescents likely to experience chronic loneliness should be given attention and support, including approaches to enhance their social connectedness. This may help reduce their loneliness and prevent future suicidality [37–39]. Unlike the findings by Schinka et al. [13], which found no elevated risk for suicidality among those whose loneliness decreased with age, we found that adolescents in the “moderate–increasing” and “moderate–decreasing” groups were at higher risk for suicidality by age 16 compared to those in the “consistently low” group. Although future studies replicating the observed association between loneliness trajectories and suicidality will further strengthen available evidence, our results suggest that adolescents who are likely to experience loneliness at any degree, not limited to those with increased or chronic loneliness, may benefit from interventions aimed at reducing loneliness.

Our study has many strengths, including the use of a large population-based cohort among contemporary adolescents in Tokyo, repeated measurement of loneliness across early-to-mid adolescence, and rich variables measured in the TTC, which allowed us to examine the role of important predictors adjusted for various covariates with little recall bias.

However, our study also had several limitations. First, loneliness was measured using a single item from the Short Mood and Feelings Questionnaire. However, this single-item measure correlates well with multi-item scales, such as the University of California Los Angeles Loneliness Scale [25, 26]. Furthermore, it has been used in other large-scale studies [14, 30] and was shown to capture the context of social relationships [14]. Second, although our sample was relatively large for a study on adolescent loneliness [9–13], only 24 participants were classified into the “consistently high” group. Therefore, the estimates presented for this group should be interpreted with caution. Relatedly, owing to the aim of this study and the larger sample size required to provide robust estimates, we decided not to investigate potential sex differences in our analysis, which could be a target of future research. Third, loneliness and our outcomes were measured based on self-reports, which may be prone to social desirability bias [40]. However, indicators of internal traits, such as loneliness or suicidal ideation, have been reported to be most accurately measured by self-ratings [41]. Finally, causal relationships cannot be inferred owing to the study’s observational design. In addition, the association between some predictors and loneliness could be bidirectional. For example, while bullying victimization was a predictor of loneliness, feeling lonely may increase vulnerability to bullying victimization and amplify subsequent emotional distress [20, 42]. Nevertheless, our demonstration of an association between important contextual factors and loneliness trajectories, and their association with suicidality by age 16, provides essential evidence for preventive interventions.

## Conclusion

While most participants had a low risk of experiencing loneliness across early-to-mid adolescence, around one-fifth had a moderate risk of experiencing loneliness at age 10, which increased with age for some, and a small group experienced loneliness throughout. Adolescents who experienced chronic loneliness were at the greatest risk for suicidality by age 16. However, despite the risk being lower, those who experienced moderate loneliness at age 10 were also at increased risk. These findings emphasize the need to raise awareness of adolescent loneliness at any degree to prevent adolescent suicidality. Adolescent loneliness should be monitored continuously, and professionals working with adolescents who report loneliness should offer timely support, especially for those reporting chronic loneliness. Our findings on the predictors of adolescent loneliness trajectories indicate that approaches to tackle bullying in early adolescence and support parents with high psychological distress may help reduce adolescent loneliness, including increased or chronic loneliness. Together, these approaches may help reduce adolescent loneliness and mitigate adolescent suicidality.

## Supplementary Information

**Additional file 1:** Model fit criteria for 2 to 5 class models for adolescent loneliness trajectories.

**Additional file 2:** Descriptive characteristics of the imputed sample.

**Additional file 3:** Descriptive characteristics of the sample by adolescent loneliness trajectories.

**Additional file 4:** Proportion of adolescents in each loneliness category at each wave, by identified trajectories.

## Data Availability

The data supporting this study’s findings are available on request from the corresponding author. The data are not publicly available owing to privacy concerns.

## Abbreviations

aRRR: Adjusted relative risk ratio
CI: Confidence interval
RR: Relative risk
TTC: Tokyo Teen Cohort
