# Supplementary material for: Predictors of chronic loneliness during adolescence: a population-based cohort study
Source: Child Adolesc Psychiatry Ment Health. 2022 Dec 21;16:107. doi: 10.1186/s13034-022-00545-z (PMC9769463; doi:10.1186/s13034-022-00545-z)
Supplement: Supplementary file 1 — Additional file 1: Model fit criteria for 2 to 5 class models for adolescent loneliness trajectories. [file 13034_2022_545_MOESM1_ESM.docx]

Table S1 Model fit criteria for 2 to 5 class models for adolescent loneliness trajectories

| Full sample | 2 class model | | 3 class model | | 4 class model | | 5 class model | |
| --- | --- | --- | --- | --- | --- | --- | --- | --- |
| N | 3,165 | | 3,165 | | 3,165 | | 3,165 | |
| AIC | 10732.44 | | 10703.96 | | 10683.72 | | 10676.40 | |
| Adjusted BIC | 10755.50 | | 10738.55 | | 10729.84 | | 10734.05 | |
| Entropy | 0.58 | | 0.58 | | 0.56 | | 0.62 | |
| Vuong-Lo-Mendell-Rubin test | 0.000 | | 0.000 | | 0.003 | | 0.000 | |
| Class membership | n | % | n | % | n | % | n | % |
| Class 1 | 2,727 | 86.2 | 115 | 3.6 | 508 | 16.1 | 13 | 0.4 |
| Class 2 | 438 | 13.8 | 367 | 11.6 | 185 | 5.8 | 26 | 0.8 |
| Class 3 |  |  | 2,683 | 84.8 | 24 | 0.8 | 522 | 16.4 |
| Class 4 |  |  |  |  | 2,448 | 77.3 | 189 | 6.0 |
| Class 5 |  |  |  |  |  |  | 2,415 | 76.3 |
