# Supplementary material for: Predictors of chronic loneliness during adolescence: a population-based cohort study
Source: Child Adolesc Psychiatry Ment Health. 2022 Dec 21;16:107. doi: 10.1186/s13034-022-00545-z (PMC9769463; doi:10.1186/s13034-022-00545-z)
Supplement: Supplementary file 2 — Additional file 2: Descriptive characteristics of the imputed sample. [file 13034_2022_545_MOESM2_ESM.docx]

Table S2 Descriptive characteristics of the imputed sample (N = 3,165)

|  |  | % | 95%CI |
| --- | --- | --- | --- |
| Sex |  |  |  |
|  | Boy | 53.1 | 51.3–54.8 |
|  | Girl | 46.9 | 45.2–48.7 |
| Parental origin |  |  |  |
|  | Japanese | 97.7 | 97.2–98.2 |
|  | Non–Japanese | 2.3 | 1.8–2.8 |
| Low parental education^a^ |  |  |  |
|  | No | 83.3 | 82.0–84.6 |
|  | Yes | 16.7 | 15.4–18.0 |
| Low household income^b^ |  |  |  |
|  | No | 89.2 | 88.1–90.3 |
|  | Yes | 10.8 | 9.7–11.9 |
| Single parent household |  |  |  |
|  | No | 95.0 | 94.2–95.8 |
|  | Yes | 5.0 | 4.2–5.8 |
| Child chronic health condition |  |  |  |
|  | No | 89.6 | 88.5–90.6 |
|  | Yes | 10.4 | 9.4–11.5 |
| Child cognitive delay^c^ |  |  |  |
|  | No | 95.3 | 94.5–96.0 |
|  | Yes | 4.7 | 4.0–5.5 |
| Bullying victimisation |  |  |  |
|  | No | 73.3 | 71.8–74.9 |
|  | Yes | 26.7 | 25.1–28.2 |
| Parental psychological distress^d^ |  |  |  |
|  | No | 95.0 | 94.2–95.8 |
|  | Yes | 5.0 | 4.2–5.8 |

^a^Defined as the respondent parent having completed a higher or lower qualification than high school.

^b^Defined as a household income below 4,000,000 Yen (approximately $30,000 US Dollars).

^c^Defined as intelligence quotient below 85.

^d^Defined as scoring above 10 on Kessler Psychological Distress Scale (K6+)
