# Supplementary material for: Predictors of chronic loneliness during adolescence: a population-based cohort study
Source: Child Adolesc Psychiatry Ment Health. 2022 Dec 21;16:107. doi: 10.1186/s13034-022-00545-z (PMC9769463; doi:10.1186/s13034-022-00545-z)
Supplement: Supplementary file 3 — Additional file 3: Descriptive characteristics of the sample by adolescent loneliness trajectories. [file 13034_2022_545_MOESM3_ESM.docx]

Table S3 Descriptive characteristics of the sample by adolescent loneliness trajectories (N = 3,165)

|  | Consistently low  (n =2,448,77.3%) | | Moderate-decreasing  (n = 185, 5.8%) | | Moderate-increasing  (n = 508, 16.1%) | | Consistently high  (n = 24, 0.8%) | |
| --- | --- | --- | --- | --- | --- | --- | --- | --- |
|  | n | % | n | % | n | % | n | % |
| Sex |  |  |  |  |  |  |  |  |
| Boy | 1,378 | 56.3 | 93 | 50.3 | 198 | 39.0 | 11 | 45.8 |
| Girl | 1,070 | 43.7 | 92 | 49.7 | 310 | 61.0 | 13 | 54.2 |
| Parental origin |  |  |  |  |  |  |  |  |
| Japanese | 2,399 | 98.0 | 182 | 98.4 | 489 | 96.3 | 23 | 95.8 |
| Non–Japanese | 49 | 2.0 | 3 | 1.6 | 19 | 3.7 | 1 | 4.2 |
| Low parental education^a^ |  |  |  |  |  |  |  |  |
| No | 2,065 | 84.4 | 150 | 81.1 | 400 | 78.7 | 18 | 75.0 |
| Yes | 380 | 15.6 | 35 | 18.9 | 108 | 21.3 | 6 | 25.0 |
| Low household income^b^ |  |  |  |  |  |  |  |  |
| No | 2,118 | 89.8 | 157 | 88.2 | 422 | 87.0 | 20 | 83.3 |
| Yes | 239 | 10.2 | 20 | 11.8 | 61 | 13.0 | 4 | 16.7 |
| Single parent household |  |  |  |  |  |  |  |  |
| No | 2,329 | 95.1 | 175 | 94.6 | 481 | 94.7 | 22 | 91.7 |
| Yes | 119 | 4.9 | 10 | 5.4 | 27 | 5.3 | 2 | 8.3 |
| Child chronic health condition |  |  |  |  |  |  |  |  |
| No | 2,211 | 90.5 | 155 | 83.8 | 443 | 87.7 | 20 | 83.3 |
| Yes | 233 | 9.5 | 30 | 16.2 | 62 | 12.3 | 4 | 16.7 |
| Child cognitive delay^c^ |  |  |  |  |  |  |  |  |
| No | 2,334 | 95.4 | 176 | 95.1 | 484 | 95.3 | 19 | 79.2 |
| Yes | 112 | 4.6 | 9 | 4.9 | 24 | 4.7 | 5 | 20.8 |
| Bullying victimisation |  |  |  |  |  |  |  |  |
| No | 1,848 | 75.7 | 123 | 66.5 | 332 | 65.7 | 10 | 41.7 |
| Yes | 591 | 24.3 | 62 | 33.5 | 173 | 34.3 | 14 | 58.3 |
| Parental psychological distress^d^ |  |  |  |  |  |  |  |  |
| No | 2,331 | 95.7 | 179 | 96.8 | 461 | 91.8 | 19 | 79.2 |
| Yes | 105 | 4.3 | 6 | 3.2 | 41 | 8.2 | 5 | 20.8 |

Observed numbers and imputed percentages are shown. N varies due to missing values. All variables were measured at age 10 (baseline).

^a^Defined as the respondent parent having completed a higher or lower qualification than high school.

^b^Defined as a household income below 4,000,000 Yen (approximately $30,000 US Dollars).

^c^Defined as intelligence quotient below 85.

^d^Defined as scoring above 10 on the Kessler Psychological Distress Scale (K6+)
