# Supplementary material for: Predictors of chronic loneliness during adolescence: a population-based cohort study
Source: Child Adolesc Psychiatry Ment Health. 2022 Dec 21;16:107. doi: 10.1186/s13034-022-00545-z (PMC9769463; doi:10.1186/s13034-022-00545-z)
Supplement: Supplementary file 4 — Additional file 4: Proportion of adolescents in each loneliness category at each wave, by identified trajectories. [file 13034_2022_545_MOESM4_ESM.docx]

Table S4 Proportion (%) of adolescents in each loneliness category at each wave, by identified trajectories

| Trajectory groups | | Category 1  (‘No’) | Category 2 (‘Sometimes’) | Category 3 (‘True’) |
| --- | --- | --- | --- | --- |
| Consistently low | Age 10 | 90.2 | 8.0 | 1.8 |
|  | Age 12 | 96.8 | 2.6 | 0.5 |
|  | Age 14 | 97.0 | 2.5 | 0.5 |
|  | Age 16 | 91.5 | 7.0 | 1.5 |
| Moderate- decreasing | Age 10 | 49.5 | 35.9 | 14.6 |
|  | Age 12 | 58.4 | 30.9 | 10.7 |
|  | Age 14 | 96.4 | 3.0 | 0.6 |
|  | Age 16 | 100 | 0 | 0 |
| Moderate-increasing | Age 10 | 60.1 | 29.8 | 10.0 |
|  | Age 12 | 63.5 | 27.7 | 8.8 |
|  | Age 14 | 58.5 | 30.9 | 10.7 |
|  | Age 16 | 44.4 | 38.2 | 17.4 |
| Consistently high | Age 10 | 15.2 | 36.4 | 48.4 |
|  | Age 12 | 11.9 | 32.7 | 55.4 |
|  | Age 14 | 6.6 | 23.1 | 70.2 |
|  | Age 16 | 2.6 | 10.9 | 86.5 |
